# Socio-economical factors that influence the perception of quality of life in patients with osteoporosis

**Published:** 2015

**Authors:** M Abobului, F Berghea, V Vlad, A Balanescu, D Opris, V Bojinca, D Predeteanu, R Ionescu

**Affiliations:** * “Sf. Maria” Clinical Hospital, Bucharest, Romania

**Keywords:** socio-economical factors, quality of life, osteoporosis, health status

## Abstract

The appearance of osteoporosis in elders and the growth of the frequency which it is diagnosed with as we approach patients who are older and older, makes this health problem very important in the societies in which a high number of persons reach old age. These societies, usually belonging to economically advanced jurisdictions, are the first interested in the way health expenses can balance the benefits of the quality of life acquired in these groups of population.

The evaluation of the quality of life has become a very important process, which still raises methodological problems to the researchers.

The aim of this study was to analyze to what extent the factors involved in defining the quality of life by the patients modified according to the existence of osteoporosis as a defined but also as a perceived disease, as far as it is considered a serious or less serious affection by each patient.

210 female patients participated in the study.

The statistical analysis was done by using SPSS 22.0 (IBM Corp. – U.S.A.). p < 0,05 was used as a limit for the statistical significance. Descriptive and analytical analyses were made by following Pearson correlation index in cases of normal distributions, the comparison between groups was made by using t-Student test, respectively chi square test in the cases which required its use.

The current study highlights a direct relationship between the quality of life, as it is perceived by the patients, and the quality of the health status, which is more important than the relationship between the quality of life and the other objectives measured by WHOQOL scale. This study also shows that for the Romanian patient diagnosed with osteoporosis, who is enclosed in the age limits of this study, the health status represents the main driver of monitoring the quality of life.

## Introduction

The appearance of osteoporosis in elders and the growth of the frequency which it is diagnosed with as we approach patients who are older and older, makes this health problem very important in the societies in which a high number of persons reach old age [**1**,**2**]. These societies, usually belonging to economically advanced jurisdictions, are the first interested in the way health expenses can balance the benefits of the quality of life acquired in these groups of population. 

The evaluation of the quality of life has become a very important process [**3**], which still raises methodological problems to the researchers. First, the question regarding the validity of the tools of the quality of life is posed, although the same tools are used in larger groups of population, including youngsters, adults but also elders. What should be taken into account is that those items which represent elements with an important value in the quality of life of youngsters do not have the same value for elders and vice versa [**4**,**5**]. The current scales used for the evaluation of the quality of life are mainly represented by SF-36 scale (which was privately developed for the access to the way of calculating the indexes and for the publication rights of the results, a financial contribution being necessary [**6**,**7**]) and WHOQOL scale, developed by the World Health Organization, offered for free for research purposes. This scale, WHOQOL, was developed at the beginning of the 90’s and it tried to validate itself at the level of different cultures with the declared objective of comparing the information obtained in different population areas [**8**,**9**]. WHOQOL-100 was developed as a multidimensional generic tool whose intention is to measure the quality of life (QOL), both in patients with health problems and in healthy populations. In 1995, the scale was reduced to only 26 items, starting from the initial version of 100 items, and this new evolution was called WHOQOL- BREF [**10**,**11**]. The scale is available in over 50 languages and it was validated for multicultural use. The WHOQOL domains are the following: physical (7 items), psychological (6 items), social relationships (3 items) and relationships in the environment (8 items). Moreover, there are also two global questions which try to evaluate the satisfaction regarding the health status and the quality of life. Each item is evaluated according to Likert scale in 5 points. In addition, each item is evaluated by taking into consideration the last two weeks of the participant. Higher values show a better quality of life and lower values show a worse quality of life. However, there are some exceptions, in questions 3, 4, and 26, which should be recoded. As mentioned before, the evaluation of the quality of life has to use of some tools in which the evaluated elements should totally reflect in the resulted scores. The internal and external validity, the discriminant power and feasibility of administrating such a questionnaire have been tested for the initial questionnaire as for each version translated in the existent languages. 

Taking into account the wider extension of SF-36 questionnaire and the relatively late appearance of WHOQOL variant, the question to what extent the two questionnaires show in fact the same status of the quality of life, was posed. A number of existent studies compared the results of applying the two questionnaires in various groups of persons, analyses being realized according to age, sex, ethnic group, social status and income status. These evaluations largely demonstrated that the two questionnaires, SF-36 and WHOQOL 100 overlap as far as indicating the way patients, respectively the respondents, appreciate their quality of life. 

When talking about PROs (Patient-Reported Outcomes), we have access to a specialty literature in which the number of publications increases exponentially each year. The explanation resides in the fact that in advanced societies, the elders are mainly interested in the health status and in the quality of life and less interested in the level of the earnings, the percent of these persons being increasingly higher. PROs have become a very important subject especially in the pathology that affects the elders, osteoporosis being in this case a very good example. There is an overlapping of the terms between Quality of Life (QOL) and the Health-Related Quality of Life (HRQOL), Health Status (HS) and, not least, the Subjective Well-Being (SWB). A special attention should be given to the separation of these notions because they are not totally interchangeable, but, despite this warning, many authors use them this way. In a meta-analysis realized by Gill and Feinstein on 75 articles which presupposed the use of QOL measurement tools, only 15 took into account the definition of QOL and even a lower number of articles justified the choice of the QOL measurement tool in that certain study [**12**]. It seems that none of these articles managed to clearly distinguish the differences between SWB, QOL and HRQOL, which created confusion both for the reader but mostly for the researcher who tried to develop his further studies based on the already published results. 

## Aim

The aim of this study was to analyze to what extent the factors involved in defining the quality of life by the patients modified according to the existence of osteoporosis as a defined but also as a perceived disease, as far as it is considered a serious or less serious affection by each patient. 

## Methods

***Participants in the study and the collection of data***

210 female patients participated in the study. They were identified in the Rheumatology Clinic of “Sfanta Maria” Hospital according to the following algorithm: patients known to suffer from osteoporosis, in whom the DXA examination confirmed the presence of osteoporosis or patients with a high risk of osteoporosis (for example suffering from early menopause, being under an aggressive and prolonged corticosteroid treatment, being under a prolonged metotrexat treatment, with presence of other comorbidities which generate osteoporosis), were included in the study. In the first stage, these patients were measured the bone mineral density and, on this occasion, we were able to identify both patients with a clear diagnosis of osteoporosis and patients in whom the bone mineral density was below the international acceptance level for defining this disease (standard deviation of -2,5). Then, a questionnaire was developed based on the questions included in the Romanian variant of the WHOQOL questionnaire, while also adding demographical and other supplementary data which helped positioning the patient in a certain socio-economical category. Data regarding the treatments most often used in Rheumatology, known to generate or on the contrary protect against osteoporosis, were collected. The same Likert scale as in the original WHOQOL variant, with numbers from 1 to 5, was used. The questions were the following: 

1. How satisfied are you of your health status? 

2. How easy do you accomplish the things you propose? 

3. How sure are you of your future? 

4. How clean and healthy is your work place? 

5. Are you satisfied with your income? 

6. How easy do you have access to the medical services? 

7. How easy can you walk for 100 meters? 

8. Do you have a various diet? 

9. How many glasses of wine do you drink daily?

10. How many packs of cigarettes do you smoke daily? 

11. Did you have a stable job in the last 10 years? 

12. How stressed are you?

13. How many true friends do you have? 

14. How much of your income goes on medication? 

15. How much of your income goes on food? 

16. Have thought about death in the last week? 

In questions regarding the amount of money spent on medication and on food the answers ranged between the following percentages: in question regarding the amount of money spent on medication the values were: 1) over 30%, 2) 20-30%, 3) 10-20%, 4) below 10%, 5) 0 or I do not buy the medication. In question regarding the amount of money spent on food the values were: 1) over 40%, 2) 30-40%, 3) 20-30%, 4) below 20% or I do not buy the food.

***Statistical analysis***

The statistical analysis was done by using SPSS 22.0 (IBM Corp. – U.S.A.). p < 0,05 was used as a limit for the statistical significance. Descriptive and analytical analyses were made by following Pearson correlation index in cases of normal distributions, the comparison between groups was made by using t-Student test, respectively chi square test in the cases which required its use. 

## Results

210 female patients were included in the study, among whom 77% met the DXA criterion for the definition of osteoporosis. As far as the treatment of these patients is concerned, 73 were under a treatment for osteoporosis (osteoform or inhibiting the bone destruction). 79,5% were under a calcium treatment (the frequency and quantity did not matter), 22,8% were under a corticoids treatment, 42,4% were under a biological therapy, 80,9% were under a nonsteroidal anti-inflammatory treatment ± aspirin and 38,6% were under a hypolipemiant/ antiaggregant/ antiarrhythmic therapy. Among the patients who were under a corticosteroids treatment, only 43,6% were also under an antiosteoporotic treatment. The age of the patient varied between 25 and 87 years, with a medium of 64,69 years and a standard deviation of 11,493. Patients who had been diagnosed with osteoporosis had this disease for approximately 5,66 years (standard deviation of 3,67). 

**Fig. 1:**
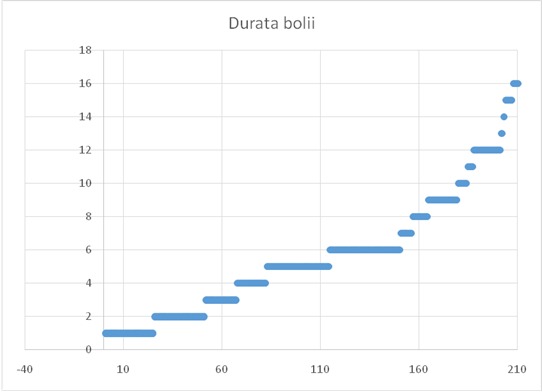
Duration of the disease

As far as Pearson correlations are concerned, it should be noticed that the patients appreciate their quality of life more concomitantly with a risen adherence to the treatment (Pearson correlation index of 0,494, p < 0,001). 

**Fig. 2:**
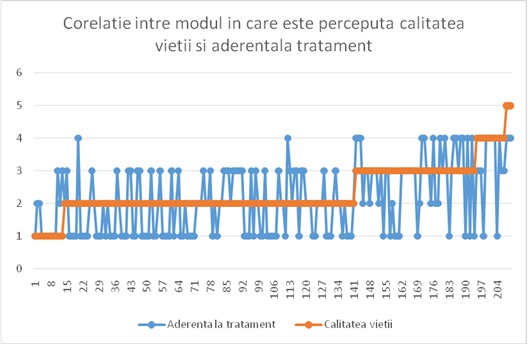
Correlation between the perception of quality of life and adherence to treatment

The correlation between the quality of life and the adherence to the doctor’s advice goes in the same direction (correlation index of 0,386 when p < 0,001). What is interesting is that a very good correlation (correlation index 0,591 when p < 0,001) appeared between the way patients appreciate their quality of life and the way they are satisfied by their health status. 

**Fig. 3:**
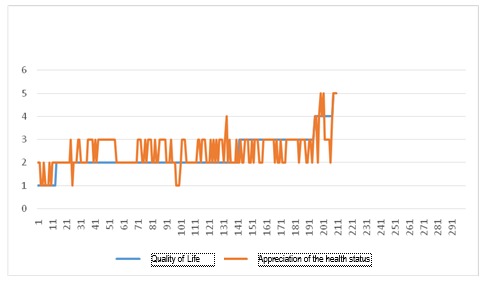
Correlation between the perception of quality of life and the health status

**Fig. 4:**
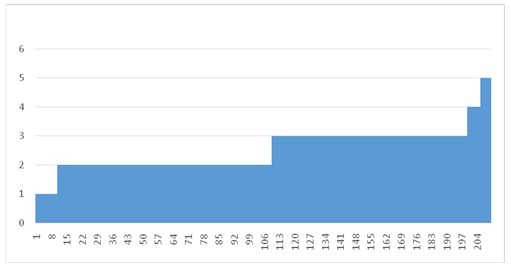
Appreciation of the health status

**Fig. 5:**
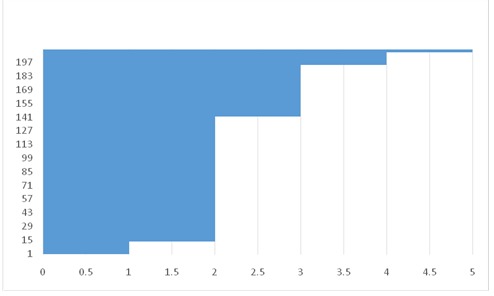
Appreciation of the quality of life

This makes us think that the quality of health has an important share in the definition of the quality of life. As far as the capacity of achieving the objectives is concerned, the patients who consider that they have a satisfying quality of life are the same who consider they can easily achieve the objectives (Pearson index of 0,285, p < 0,001).

The correlation between the quality of life and the available amount of money is positive, but very weak (Pearson index of 0,163, p < 0,05). This means that the patients connect the quality of life with the quality of health more than with the access to high financial resources. As far as the satisfaction regarding the health status is concerned, a positive but low degree of correlation was noticed as compared with the time since the patient was diagnosed with osteoporosis (Pearson index of 0,168 when p < 0,05), with the adherence to the treatment (Pearson index of 0,311 when p < 0,001), the compliance with the doctor’s advice (Pearson index of 0,232 when p < 0,001), but also a superior correlation level with regard to the way the patient appreciates the quality of life as far as the following aspects are concerned: how easy do you accomplish the things you propose? (Pearson index of 0,419, p < 0,001), how sure are you of your future? (Pearson index of 0,222, p < 0,001) and especially: are you satisfied with your income? (Pearson index of 0,234, p < 0,001). These aspects make us conclude that the health status and not the quality of life is considered the main determinant factor in achieving the objectives and, on the other side, the health status and not the quality of life represents the constant indicator of a long future and a quality life. 

**Fig. 6:**
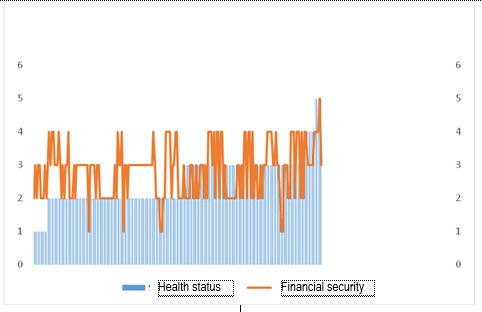
Correlation between the health status and the financial security (weak)

**Fig. 7:**
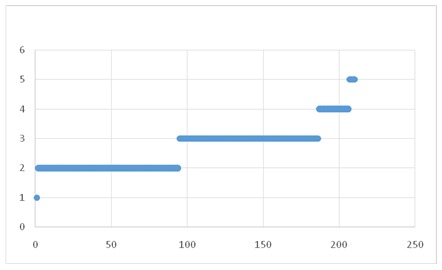
Security of the future

**Fig. 8:**
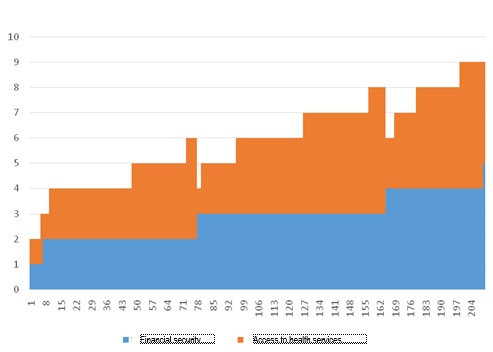
Financial security vs. access to health services

**Fig. 9:**
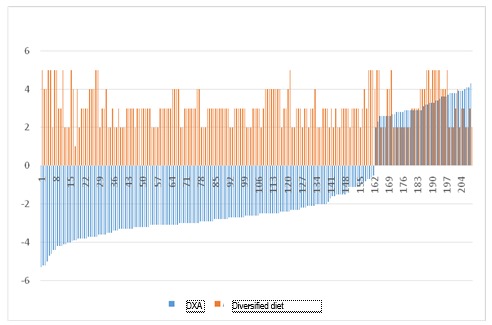
DXA value vs. quality of diet

**Fig. 10:**
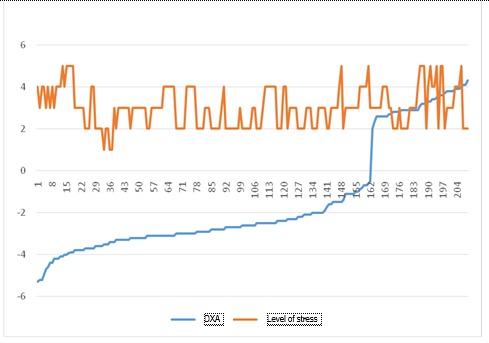
Level of stress vs. DXA

We can notice the difference between the quality of life and the quality of health status reported to the income of the person. The patients with higher incomes are the same who consider that their health status is better and not the ones with higher incomes versus a better quality of life. What is interesting to notice is the fact that even the patients who consider they are balanced from the financial point of view, they adhere better to the doctor’s indications, this correlation being statistically significant but very weak (Pearson index of 0,137, when p < 0,05). As far as the opinions or the ideas of suicide are concerned, stronger correlations were noticed, as expected, in the patients low on money (Pearson index of 0,289, p < 0,001), in patients who do not have an easy access to medical services (Pearson index of 0,241, p < 0,001), in patients who have a difficulty in walking (Pearson index of 0,275, p < 0,001), in patients who do not have a diversified diet (Pearson index of 0,380, p < 0,001), in patients who consume a large amount of alcohol (Pearson index of 0,250, p < 0,001), in patients who are very stressed (Pearson index of 0,206, p < 0,001), in patients who have a few friends (Pearson index of 0,345, p < 0,001), in patients who use a high percent of their income on medication (Pearson index of 0,222, p < 0,001) or on food (Pearson index of 0,322, p < 0,001), as well as in patients who consider that are globally low on money (Pearson index of 0,289, p < 0,001). Other significant correlations with the suicide idea, however without having a special statistical power, were with the quality of life (Pearson index of 0,156, p < 0,05), the ability of meeting targets in life (Pearson index of 0,155, p < 0,05) and the health status (Pearson index of 0,177, p < 0,05).

**Fig. 11:**
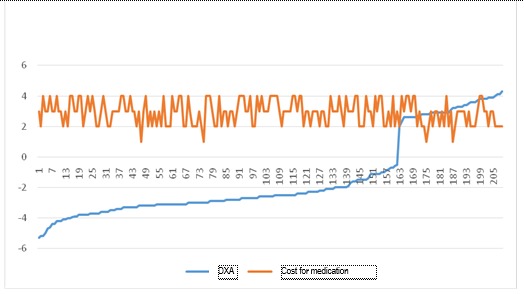
Correlation between DXA and the share of medication in the monthly expences

## Discussions

The current study highlights a direct relationship between the quality of life, as it is perceived by the patients, and the quality of the health status, which is more important than the relationship between the quality of life and the other objectives measured by WHOQOL scale. This study also shows that for the Romanian patient diagnosed with osteoporosis, who is enclosed in the age limits of this study, the health status represents the main driver of monitoring the quality of life. 

It can be noticed that as patients adhere to the treatment and to the doctor’s indications, they consider that both the health status and the quality of life are better. It remains to be determined to what extent these patients have a particular psychological profile which makes them be optimistic about the quality of life and the health status and adhere independently of other factors to the doctor’s treatment indications. 

The relationship between the financial capacity and the access to the health services is highlighted by this study: even when most of the health services specific for osteoporosis are free, patients consider this disease is treated in a way which requires a financial supplement. Moreover, we can observe that depression is not mainly connected to the way quality of life or quality of health are perceived, but mostly by external factors, respectively the access to a various diet or an adequate financial level. This is surprising as long as an adequate financial level is not considered the main driver of the access to health services. It still remains to be seen to what extent depression in this kind of patients is connected to other entities than the main disease, osteoporosis. 

Patients who consider they are deeply anchored socially, respectively they have many real friends are also those who consider that their health status and the quality of life are maximum. On the other side, patients who consider they are stressed at home or at the working place are also those who admit a weaker quality of life and quality of health. 

To our knowledge, this study is the first to evaluate the patients with osteoporosis in the context of the socio-economical indicators. The results are interesting and they represent a challenge both for the providers of the health services and the executives of health politics as far as the optimization of resources allocation are concerned, so that the patients are aware both of the services received and of the obtained results which reflect in the quality of life and the quality of health. 

A limit to this study is represented by the fact that it did not include other pathologies and it only compared patients with osteoporosis defined by DXA score with patients without osteoporosis, but with a high risk of developing osteoporosis, in the absence of a control group with other pathologies and similar demographical characteristics. In the future, such a comparison should also be realized, in order to see to what extent, for example, a chronic disease differs from an acute disease of a higher or lower intensity, as far as the impact on quality of life and quality of health are concerned. Moreover, it is important to think to what extent the access to generous financial resources is considered important in a chronic entity as compared to an acute pathological entity. It still remains to be seen if other studies developed in the future will also answer to these challenges. 

**Conflict of interest**

None. 

The article is part of the “Introduction” and “Special Part” of the doctoral thesis of the corresponding author, Abobului Mihai, MD, PhD student.

## References

[1] (2000). Osteoporosis prevention, diagnosis, and therapy. NIH Consensus Statement Online.

[2] (1994). Assessment of fracture risk and its application to screening for postmenopausal osteoporosis: report of a World Health Organization Study Group. WHO Technical Report Series No. 843.

[3] McNeil WH (1998). Plagues and Peoples.

[4] Guillemin F, Bombardier C, Beaton D (1993). Cross-cultural adaptation of health-related quality of life measures: literature review and proposed guidelines. J Clin Epidemiol.

[5] Sartorius N, Kuyken W (1994). Translation of health status instruments. In: Orley K, Kuyken W (eds). Quality of Life Assessment: International Perspectives.

[6] Wagner AK, Gandek B, Aaronson NK, Acquadro C, Alonso J, Apolone G (1998). Cross-cultural comparisons of the content of SF-36 translations across 10 countries: Results from the IQOLA Project. International Quality of Life Assessment. J Clin Epidemiol.

[7] ABS (1995). National Health Survey: SF-36 Population Norms, Australia. ABS Catalogue No. 4399.0.

[8] (1995). WHOQoL Group. The World Health Organization Quality of Life Assessment (WHOQOL): position paper from the World Health Organization. Soc Sci Med.

[9] (1998). WHOQoL Group. Development of the World Health Organization WHOQOL-BREF Quality of Life Assessment. Psychol Med.

[10] (1998). WHOQoL Group. The World Health Organization Quality of Life Assessment (WHOQOL): Development and general psychometric properties. Soc Sci Med.

[11] (1996). WHOQoL Group. WHOQOL-Bref: Introduction, Administration, Scoring and Generic Version of the Assessment.

[12] Gill TM, Feinstein AR (1994). A critical appraisal of the quality of quality-of-life measurements. JAMA.

